# Effect of intermittent extracellular low-pH environment on human umbilical vein endothelial cell activity

**DOI:** 10.1371/journal.pone.0332673

**Published:** 2025-09-16

**Authors:** Ryota Nishida, Tomoaki Fukui, Kenichi Sawauchi, Yohei Kumabe, Hyuma Kondo, Yuya Yamamoto, Kyohei Takase, Ryo Yoshikawa, Takahiro Niikura, Ryosuke Kuroda, Keisuke Oe

**Affiliations:** 1 Department of Orthopaedic Surgery, Kobe University Graduate School of Medicine, Kobe, Japan; 2 Department of Orthopaedic Surgery, Hyogo Prefectural Nishinomiya Hospital, Hyogo, Japan; Noorda College of Osteopathic Medicine, UNITED STATES OF AMERICA

## Abstract

Transcutaneous CO_2_ application promotes fracture healing and osteogenesis via angiogenesis. However, its molecular mechanism remains unclear. This therapy transiently decreases intra-tissue pH in the affected area to approximately 7.0 for 20 min, followed by recovery. We hypothesized that such intermittent pH changes activate endothelial cells similarly to acidic preconditioning. We aimed to investigate the response of human umbilical vein endothelial cells (HUVECs) to daily intermittent low-pH stimulation. HUVECs were cultured under three conditions: daily 20-min exposure to a low-pH medium (approximately 7.0), followed by a return to a control medium (approximately 7.4) (Change group); continuous exposure to a low-pH medium (Low pH group); constant culturing in a control medium (Control group). Cell proliferation, tube formation, migration, and protein/gene expression were assessed. Tube formation and migration were significantly enhanced in the Change group compared with those in the Control group, and tube formation was also increased in the Low pH group. Western blotting revealed upregulated expression of vascular endothelial growth factor (VEGF) and VEGF receptor 2 in the Change group. CD31 gene expression was elevated in the Low pH and Change groups on Days 1 and 7, respectively. Phosphorylated extracellular signal-regulated kinases 1 and 2 (ERK1/2) and protein kinase B (AKT) levels in the Low pH and Change groups were significantly increased compared with those in the Control, except for ERK2 in the Low pH group. The suppression of ERK1/2 or AKT by their inhibitors (U0126 and LY294002, respectively) during low-pH exposure inhibited proliferation and tube formation, indicating that the ERK1/2 mitogen-activated protein kinase (MAPK) and phosphatidylinositol-3 kinase (PI3K)/AKT pathways mediate endothelial cell activation in response to intermittent acidic stimulation. Intermittent low-pH exposure enhanced HUVEC activity, mediated by the ERK1/2 MAPK and PI3K/AKT pathways. Our findings provide insights into the mechanism by which transcutaneous CO_2_ application promotes angiogenesis and tissue repair.

## Introduction

Transcutaneous CO_2_ application is a treatment that involves applying a hydrogel to enhance CO_2_ absorption, followed by the infusion of CO_2_ gas into a sealed area of the patient’s body, such as the limb, for 20 min daily [1–5]. Our previous studies revealed that this therapy increases local blood flow and promotes angiogenesis by upregulating angiogenic factors such as vascular endothelial growth factor (VEGF) in animal models, facilitating muscle fiber type transition, early fracture healing, and osteogenesis [1–4]. Thus, the clinical application of this treatment requires validation, as it represents a promising supportive therapy for surgeries and rehabilitation across various orthopedic fields. Although angiogenesis is crucial in myogenesis and osteogenesis [6,7], the molecular mechanisms underlying the angiogenic effects of transcutaneous CO_2_ application remain poorly understood. Therefore, uncovering the *in vitro* molecular pathways mediating these effects is essential.

We previously demonstrated the feasibility of transcutaneous CO_2_ penetration *in vivo*. We prepared an airtight CO_2_ gas chamber with a 5-cm diameter hole at the bottom, covered with rat skin, to observe the permeability of CO_2_ gas through the skin. The pH of pure water, into which CO_2_ was dissolved via rat skin, decreased to approximately 7.0 within 20 min. Following transcutaneous CO_2_ application in the upper limbs of healthy volunteers, nuclear magnetic resonance (NMR) indicated that the intramuscular pH in the triceps surae muscle decreased to approximately 7.0 after 20 min of treatment and returned to its previous level within 5 min post-treatment. This implies that transcutaneous CO_2_ application causes a temporary decrease in tissue pH. We have demonstrated that this pH decrease induces the Bohr effect, enhancing microcirculation by facilitating oxygen dissociation from the hemoglobin to local tissues [5].

The physiological range of blood pH is maintained between 7.36 and 7.44. Acidosis is a condition characterized by a pH of <7.35 [8]. In various local or systemic conditions that promote angiogenesis, such as ischemia, tumor growth, wound healing, diabetic ketoacidosis, major trauma, and burns, the blood pH may drop to 7.0–6.0 [9–12]. Although acidosis reduces endothelial cell activity [13,14], short-term acidosis enhances endothelial cell function [15,16] and protects against ischemia/reperfusion injury in various tissues [17–21]. Short-term exposure to acidosis that promotes these effects is known as acidic preconditioning (APC). In the coronary system, brief acidosis without concomitant ischemia has demonstrated protective effects against ischemic reperfusion injury [17]. In addition, transient acidosis during the early reperfusion phase mediates cardioprotective effects by activating protein kinase B (AKT) and extracellular signal-regulated kinases (ERK) [18]. APC protects against ischemia-induced neuronal injury in the brain [19]. In the lungs, therapeutic hypercapnia, which induces respiratory acidosis, mitigates ischemia-reperfusion lung injury by inhibiting key transcriptional activators within the nuclear factor-kappa B pathway during inflammation [20]. In endothelial cells, APC protects coronary tissues against ischemic apoptosis [21]. Furthermore, APC enhances the angiogenic activities of endothelial progenitor cells by upregulating ERK1/2 mitogen-activated protein kinase (MAPK) and phosphatidylinositol-3 kinase (PI3K)/AKT pathways [15].

Sakai et al. reported that, as described above, transcutaneous CO_2_ application decreased intra-tissue pH to approximately 7.0, which returned to baseline within 5 min after CO_2_ discharge in *in vitro* and *in vivo* models [5]. This pH condition is comparable to that observed in APC. Therefore, we hypothesized that intermittent low-pH stimulation induced by transcutaneous CO_2_ application exerts effects similar to those of APC, promoting increased blood flow and angiogenesis. Furthermore, we assumed that these effects are mediated by the ERK1/2 and AKT pathways, as previously reported in studies on APC. However, few studies have evaluated endothelial cell activity under conditions of repeated pH changes, as those under transcutaneous CO_2_ application. To test this hypothesis, we assumed that the extracellular pH at the CO₂ treatment site was approximately 7.0 (based on our previous study [5]) and 7.4 under non-treatment conditions. Under these conditions, we aimed to explore the effects of repeated intermittent pH changes between these values on endothelial cell activity. In this study, we sought to investigate endothelial cell activity under conditions of daily 20-min intermittent low-pH stimulation and examine the relationship between endothelial activation and the ERK1/2 and AKT pathways.

## Materials and methods

### Cell culture

Human umbilical vein endothelial cells (HUVECs) were purchased from Kurabo (KE-4109, Osaka, Japan) and cultured in an endothelial cell growth medium (LEC-LL0003, Kurabo, Osaka, Japan). The medium contained the following growth factors: 5 ng/mL rhFGF, 50 µg/mL ascorbic acid, 1 µg/mL hydrocortisone hemisuccinate, 2% FBS, 10 mM L-glutamine, 15 ng/mL rhIGF-1, 5 ng/mL rhEGF, 5 ng/mL rhVEGF, and 0.75 U/mL heparin. The VEGF concentration in the medium was controlled only for the transwell migration assay. The cells were maintained in a humidified incubator at 37 °C with 5% CO_2_. The HUVECs between the third and fourth passages were analyzed.

### Low-pH medium preparation and modeling CO₂-induced extracellular acidosis

First, standard control and low-pH media were prepared, targeting pH values of approximately 7.4 and 7.0, respectively. The endothelial cell growth medium was initially placed in the CO_2_ incubator. On the following day, the medium pH was approximately 7.4; therefore, it was designated as the control medium. To establish the low-pH medium, the medium pH was lowered to approximately 7.0 through titration with 1 N HCl using a Minisart Syringe Filter (Sartorius, Germany). Each medium was stored in the CO_2_ incubator from the day before the intervention until the final day of the experiment to standardize the pH. On the first day of the intervention, the pH ranges of the control and low-pH media were 7.43–7.56 and 6.90–7.03, respectively.

Subsequently, these media were applied to culture HUVECs under three conditions. The cells subjected to the conditions designed to mimic extracellular pH changes during transcutaneous CO_2_ application were defined as the ‘Change group'. First, HUVECs were cultured in the control medium, which was replaced once daily with the low-pH medium. Next, 20 min after replacement, the low-pH medium was discarded and replaced with the control medium. This cycle was repeated once daily up to 7 days. For comparison, other HUVECs were continuously cultured in the control or low-pH medium, defined as the ‘Control’ and ‘Low pH’ groups, respectively. In these groups, the media were changed twice weekly, following the common protocol for continuous cell lines cultured in conventional medium [22]. The dish size and number of cells were determined based on the assessment. To ensure consistency across the groups except for medium pH, thawed cells were distributed into the three groups and cultured simultaneously. This procedure was repeated at least three times for each assay.

### Monitoring the medium pH in each group during cell culturing

Cells rapidly consume nutrients and release metabolites into the medium, resulting in a pH decrease [22]. To determine the daily changes in medium pH during cell incubation, we monitored the pH in each group. Cells were incubated in six-well plates at 2 × 10⁵ cells/well. In the Control and Low pH groups, the media were changed twice weekly. In the Change group, the medium was replaced daily with a low-pH medium for 20 min, followed by replacement with the control medium. For daily pH measurements, cells from each group were prepared for all time points. The experiment was conducted for 7 days, and the medium pH for each group was recorded daily (n = 2 per group).

### Proliferation assay

Cell proliferation was assessed using a cell counting kit-8 (CCK-8, Dojindo, Japan). HUVECs were seeded in 96-well plates at 4 × 10³ cells/well. All groups were cultured at 37 °C with 5% CO_2_. Daily pH changes were applied to cells in the Change group. On Days 1, 2, 4, and 7, 10 μL of a CCK-8 solution was added to the HUVECs in each well. Subsequently, the cells were incubated for 4 h at 37 °C with 5% CO_2_, and optical densities were recorded at 450 nm, representing HUVEC proliferation. For each group and time point, five wells were assessed, and the average value was calculated (n = 5 per group).

### Tube formation assay

The angiogenic activity of HUVECs was evaluated using tube formation assays. First, HUVECs were seeded in six-well plates at 2 × 10⁵ cells/well and incubated in each pH medium for 4 days. During this period, the Change group was subjected to pH modifications. Next, the HUVECs in each group were re-counted and reseeded at 4.0 × 10⁴ cells/well in the control medium onto 96-well culture plates coated with 50 μL of Matrigel (3.3 mg/mL; #356234, Corning, NY, USA). The Matrigel was plated and incubated for 3 h at 37 °C before cell seeding. Network-like structures were observed at 15 h post-seeding using a microscope. Four microscopic fields per well were randomly selected and photographed (n = 6 per group). Tube-forming ability was evaluated by measuring the total branching length and analyzing the number of branches and junctions using ImageJ software (NIH, Bethesda, MD, USA).

### Transwell migration assay

The migratory ability of the HUVECs was evaluated using transwell migration assays. Before the assay, the cells were preconditioned for 4 days in each pH-conditioned medium at 2 × 10⁵ cells/well in six-well plates. For the Change group, a daily medium change was performed for this period. After the 4-day preconditioning, the cells were harvested, re-counted, and seeded at 5 × 10⁴ cells in 500 μL of VEGF-free medium onto Matrigel-coated 8.0-μm pore polycarbonate inserts (BD Falcon®, BD Biosciences). Furthermore, 50 µL of Matrigel had been coated onto the inserts and polymerized for 3 h at 37 °C. The lower chambers were filled with 800 μL of the medium containing 20 ng/mL VEGF. The transwell migration assay was conducted over 24 h. After incubation, the upper chambers were removed, and residual Matrigel was gently wiped from the upper side of the membrane with a cotton swab. Cells that had migrated through the membrane were fixed with 4% paraformaldehyde and stained with crystal violet for 20 min. Finally, the migrated cells were imaged using a microscope, and the number of migrated cells was quantified using ImageJ software (n = 6 per group).

### Real-time reverse transcription polymerase chain reaction (RT-PCR)

HUVECs were seeded in six-well plates at 2 × 10⁵ cells/well and incubated in each pH medium for up to 7 days. Total RNA was extracted from each group on Days 1, 4, and 7 using the RNeasy Mini Kit (Qiagen, Valencia, CA, USA). The RNA was reverse-transcribed into single-stranded complementary DNA (cDNA) using a high-capacity cDNA reverse transcription kit (Applied Biosystems, Foster City, CA, USA). Real-time RT-PCR was performed in duplicate on the cDNA using an Applied Biosystems 7500 real-time PCR system and SYBR Green reagent (Applied Biosystems). The expression levels of *CD31* (Thermo Fisher Scientific, Waltham, MA, USA) were normalized to those of glyceraldehyde-3-phosphate dehydrogenase (*GAPDH*) (Thermo Fisher Scientific), a well-known housekeeping gene serving as an internal control. All results were presented as the fold-change relative to the control group, which was normalized to a value of 1 (ΔΔCt method) (n = 5 per group). The primers for the analyzed genes are presented in Table 1.

**Table 1:** Details of the primers used for amplification.

Gene	Primer sequences (5’ to 3’) (forward/reverse)
GAPDH	CCACATCGCTCAGACACCAT
	CCAGGCGCCCAATACG-
CD31	GTGCTGCAATGTGCTGTGAAT
	GTTGGCTCTGTTGAAGGCTGT

### Western blotting

Western blotting was performed to evaluate the activation of the VEGF signaling pathway induced by intermittent pH changes in the HUVECs. The primary antibodies used were rabbit anti-VEGF receptor 2 (VEGFR2, also known as KDR) (1:1000; ab315238, Abcam, Cambridge, UK); mouse anti-VEGF (1:200; sc-7269, Santa Cruz, San Diego, CA, USA); mouse anti-ERK1/2 (1:100; sc-514302, Santa Cruz, San Diego, CA, USA); mouse anti-phospho-ERK1/2 (1:200; sc-7383, Santa Cruz, San Diego, CA, USA); mouse anti-AKT (1:200; sc-5298, Santa Cruz, San Diego, CA, USA); rabbit anti-phospho-AKT (1:1000; ab38449, Abcam, Cambridge, UK); mouse anti-hypoxia-inducible factor 1 alfa (HIF1α) (1:200; sc-13515, Santa Cruz, San Diego, CA, USA); and mouse anti-β-actin (1:5000; A5441, Sigma-Aldrich, St. Louis, MO, USA) antibodies. Similar to other assessments, cells were incubated in six-well plates at 2 × 10⁵ cells/well and underwent the 4-day experiment as previously described, followed by protein extraction. Protein concentrations were measured using the Pierce BCA protein assay kit (#23227, Thermo Fisher Scientific, Waltham, MA, USA). The lysates were diluted to equal concentrations with a hypotonic buffer. Samples were separated using sodium dodecyl sulfate-polyacrylamide gradient gel electrophoresis (Biocraft, Tokyo, Japan) under reducing conditions and transferred electrically onto a blotting membrane (Amersham Biosciences Corp., Arlington Heights, IL, USA). The membranes were blocked for 1 h and incubated with primary antibodies overnight at 4 °C. Anti-mouse immunoglobulin-G horseradish peroxidase-linked (1:10000; NA-931, Cytiva, Marlborough, MA, USA) and anti-rabbit immunoglobulin-G horseradish peroxidase-linked (1:10000; NA-934, Cytiva, Marlborough, MA, USA) were used as the secondary antibodies. Images of the blots showing the protein expression were captured using the iBright™ CL1500 Imaging System (Thermo Fisher Scientific). Relative band intensities were quantified using ImageJ software (n = 5 per group). ERK and AKT activation was evaluated by measuring the relative levels of phosphorylated ERK and AKT, respectively, normalized to total ERK and AKT.

### Assessments of cell activities in the presence of ERK1/2 or AKT inhibitors

To evaluate the roles of ERK1/2 and AKT during pH changes, cell proliferation and tube formation were assessed in the presence or absence of ERK1/2 and AKT inhibitors under the conditions of the Change group. Cells were incubated in six-well plates at 2 × 10⁵ cells/well and subjected to the 4-day pH change experiments. During the 20-min exposure to the low-pH medium, the ERK1/2 (U0126, # BML-EI-282, Enzo Life Sciences, Farmingdale, NY, USA) and AKT (LY294002, # BML-ST420, Enzo Life Sciences, Farmingdale, NY, USA) inhibitors were added at 5 µM or excluded. Cells were cultured in the control medium without these inhibitors at all other times. After the 4-day pH change experiment, with or without the inhibitors, cell proliferation was measured using the CCK-8 assay (n = 5), and tube formation was assessed using the tube formation assay (n = 3), as previously described.

### Statistical analysis

The normality of the data was verified using the Shapiro–Wilk test. Differences between groups were evaluated using a one-way analysis of variance (ANOVA), followed by the Tukey–Kramer *post-hoc* test. The significance level was set to p < 0.05. Data were expressed as the mean ± standard error of the mean. All statistical analyses were conducted using EZR (Saitama Medical Center, Jichi Medical University, Saitama, Japan). A *post-hoc* power analysis was performed using G*Power 3.1.9.7. Assuming a one-way ANOVA, for a sample size of six samples in three groups and a type I error (α) of 0.05, the study was expected to achieve a power (1-β) of 0.81 for detecting an effect size of 0.82.

## Results

### During cell culturing, the medium pH initially increased and gradually decreased

**Fig 1:**
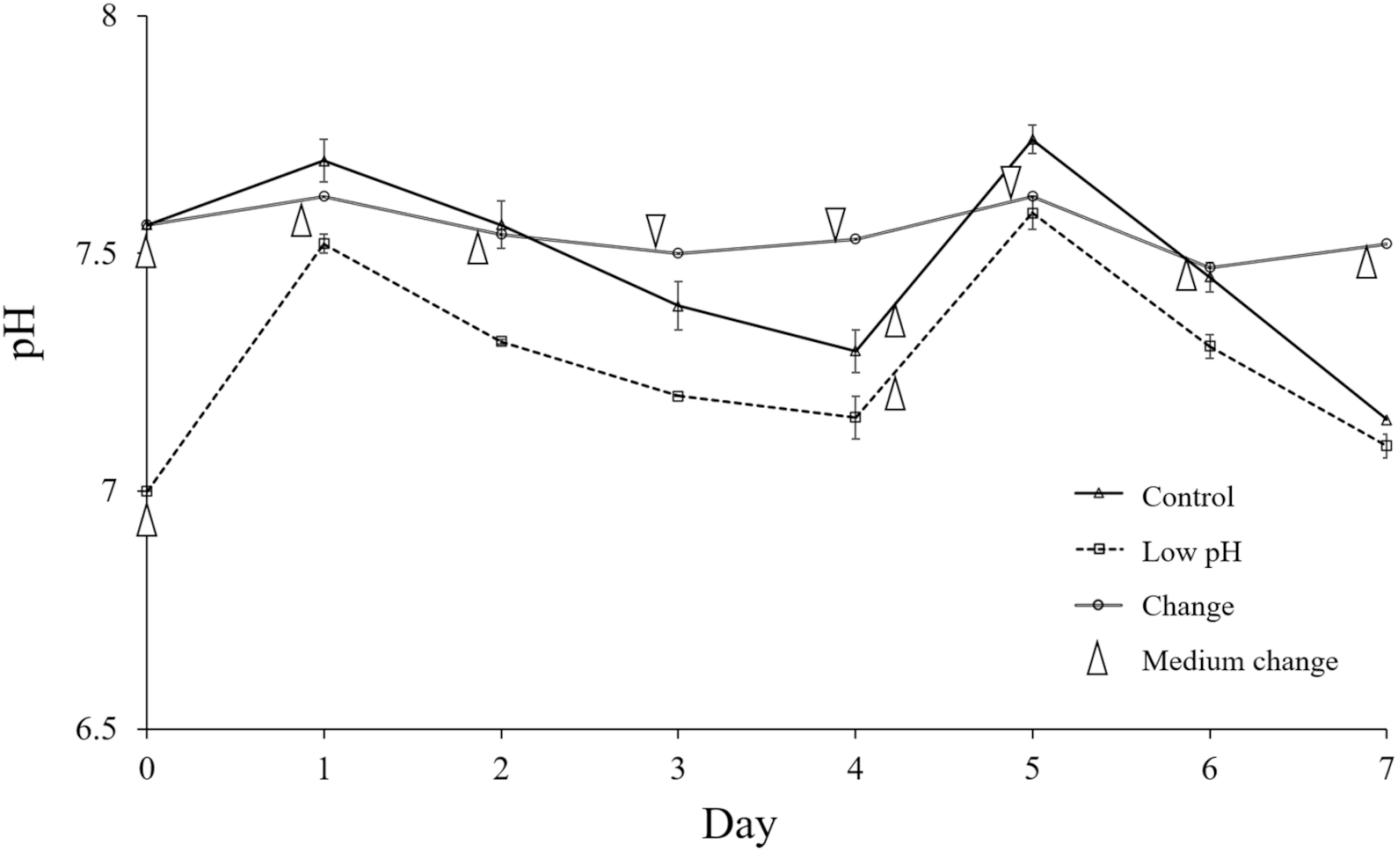
Monitoring of medium pH in each group during cell culture. The medium pH was measured in each group during cell culturing through Day 7. Data are presented as the mean ± standard error. White arrows indicate the timing of medium changes. n = 2.

In the normal and low-pH media, the medium pH increased on the first day after the medium change and gradually decreased. While the pH difference between the Control and Low pH groups became smaller than that at the initial time point, both groups maintained their relationship. In the Change group, the medium pH was limited to approximately 7.5 owing to daily medium replacement (Fig 1). These imply that the medium pH was not stably maintained over several days; however, the relationship among groups persisted. This study did not focus on the exact pH values, as the aim was to investigate the effects of environmental stimulation induced by medium replacement with a low-pH medium. Therefore, we proceeded with subsequent assessments under these conditions.

### Intermittent pH changes for 20 min daily promoted tube formation and migration abilities in the HUVECs

**Fig 2:**
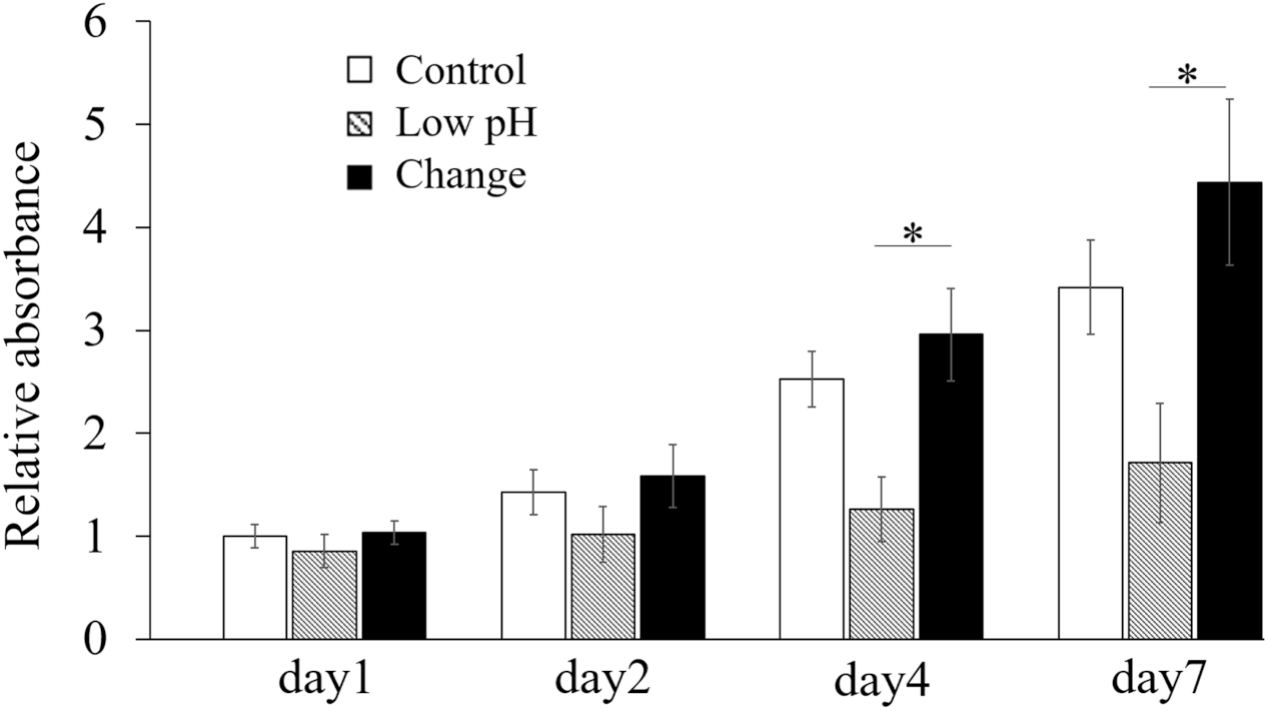
Effects of low-pH and intermittent pH changes on human umbilical vein endothelial cell (HUVEC) proliferation, as assessed using cell counting kit 8 (CCK-8). Cell proliferation was measured using the CCK-8 assay across different groups. Data are presented as the mean ± standard error. Statistical comparisons were performed using a one-way analysis of variance (ANOVA), followed by Tukey’s *post-hoc* test. n = 5; * p < 0.05.

The proliferation of the HUVECs was evaluated using the CCK-8 assay (Fig 2). The proliferation in the Low pH group was significantly reduced on Days 4 and 7 compared with that in the Change group (p = 0.013 and p = 0.025, respectively). Although the absorbance in the Change group at each time point was slightly higher than that in the Control group; however, the difference was not significant.

**Fig 3:**
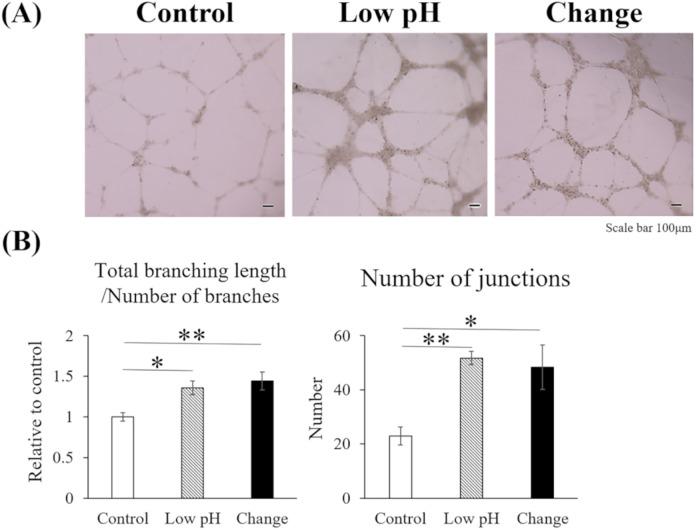
Intermittent pH changes promoted the angiogenic potential of the HUVECs in tube formation assays. The angiogenic capability of the HUVECs under different conditions was examined using tube formation assays. (A) Representative microscopic images of the three groups. Scale bar = 100 μm. (B) Quantitative analysis of the total branching length, number of branches, and number of junctions. Data are presented as the mean ± standard error. Statistical comparisons were performed using a one-way ANOVA, followed by Tukey’s *post-hoc* test. n = 6; * p < 0.05 and ** p < 0.01. Quantitative analysis was performed using ImageJ software.

The angiogenic ability of the HUVECs was assessed using a tube formation assay (Fig 3). The total branching length per number of branches and the number of junctions, calculated using ImageJ software, were significantly increased in the Low pH (p = 0.027 and p = 0.004, respectively) and Change (p = 0.006 and p = 0.010, respectively) groups compared with those in the Control group.

**Fig 4:**
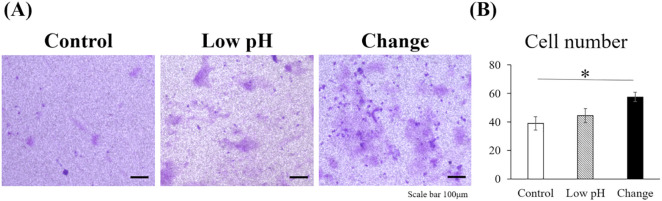
Intermittent pH changes promoted the migration activities of the HUVECs in transwell migration assays. The migratory response of HUVECs under different conditions was assessed using transwell migration assays. (A) Representative microscopic images with crystal violet staining of the three groups are shown. Scale bar = 100 μm. (B) Quantitative analysis of the number of migrated cells is presented as the mean ± standard error. Statistical comparisons were performed using a one-way ANOVA, followed by Tukey’s *post-hoc* test. n = 6; * p < 0.05. Quantitative analysis was performed using ImageJ software.

The migration ability of the HUVECs was investigated using a transwell migration assay (Fig 4). The number of cells that migrated in response to VEGF, as evaluated with ImageJ software, was significantly higher in the Change group than in the Control group (p = 0.023).

### Intermittent pH changes for 20 min daily promoted the expression of endothelial marker mRNA and angiogenic proteins

**Fig 5:**
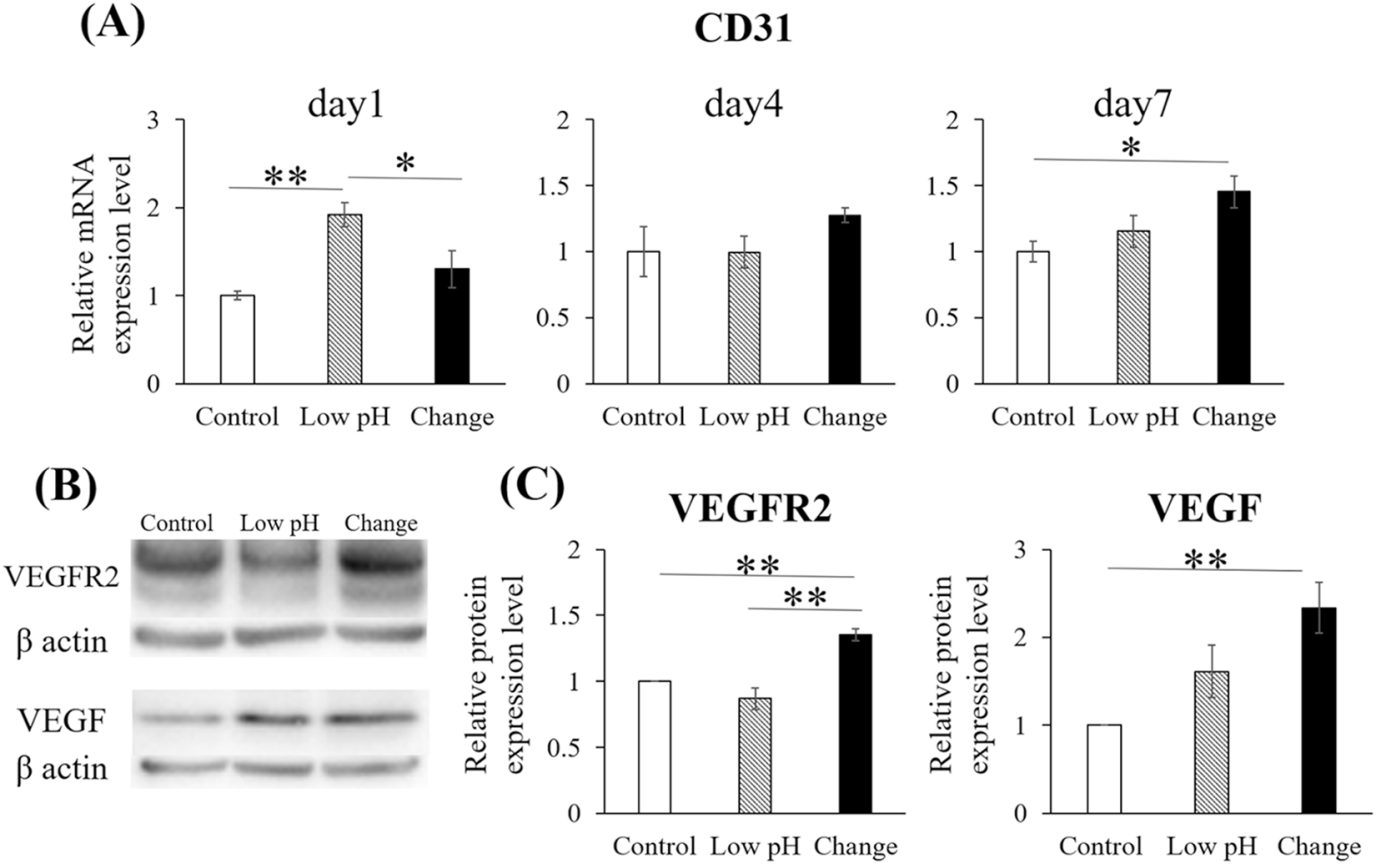
Intermittent pH changes induced the expression of CD31 mRNA, as well as those of vascular endothelial growth factor (VEGF) and VEGF receptor 2 (VEGFR2) proteins. The mRNA expression of CD31 and protein expression of VEGFR2 and VEGF were assessed. (A) The mRNA expression of CD31 on Days 1, 4, and 7 was examined using real-time reverse transcription polymerase chain reaction (RT‐PCR). (B) The protein expression levels of VEGF on Day 4 were assessed using western blotting. Representative images of the two groups are shown. (C) Quantification of protein levels relative to those of the control group was performed using ImageJ software. Data are presented as mean ± standard error. Statistical comparisons were performed using a one-way ANOVA, followed by Tukey’s *post-hoc* test. n = 5; * p < 0.05 and ** p < 0.01.

RT-PCR for *CD31* and western blotting for VEGFR2 and VEGF were performed to assess the expression of endothelial marker mRNA and angiogenic proteins, respectively. In real-time RT-PCR, *CD31* expression was assessed on Days 1, 4, and 7. On Day 1, gene expression in the Low pH group was significantly higher than that in the Control and Change groups (p = 0.002 and p = 0.028, respectively). On Day 4, no significant differences were observed among the groups. On Day 7, the expression in the Change group was significantly higher than that in the Control group (p = 0.027) (Fig 5A). In the western blotting of the proteins extracted on Day 4, VEGFR2 expression was elevated in the Change group compared with those in the Control and Low pH groups (p = 0.001 and p < 0.001, respectively). VEGF expression was significantly higher in the Change group than in the Control group (p < 0.001) (Fig 5B, 5C).

### Intermittent pH changes for 20 min daily suggested VEGF induction via ERK1/2 MAPK and PI3K/AKT pathways

**Fig 6:**
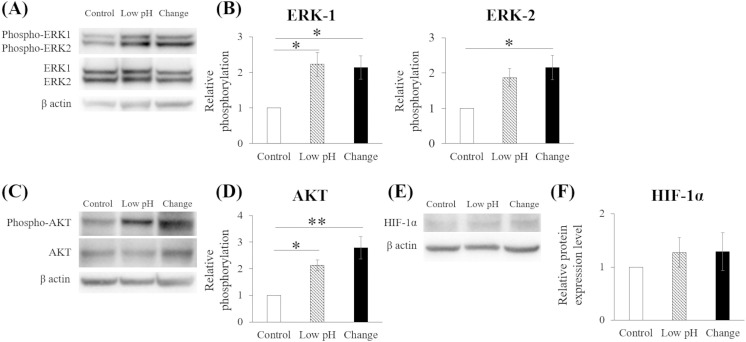
Intermittent pH changes suggest acceleration of the extracellular signal-regulated kinase 1/2 (ERK1/2) mitogen-activated protein kinase (MAPK) and phosphatidylinositol-3 kinase (PI3K)/ protein kinase B (AKT) pathways. The protein levels of phosphorylated and total ERK1/2, AKT, and hypoxia-inducible factor 1 alpha (HIF-1α) were assessed using western blotting. (A) Representative western blot images of phosphorylated and total ERK1/2. (B) Relative levels of phosphorylated ERK1/2 normalized to total ERK1/2 were evaluated using ImageJ software. (C) Representative images of phosphorylated and total AKT. (D) Relative levels of phosphorylated AKT normalized to total AKT. (E) Relative images of HIF-1α. (F) Relative protein expression levels of HIF-1α. Data are presented as mean ± standard error. Statistical comparisons were performed using a one-way ANOVA, followed by Tukey’s *post-hoc* test. n = 5, * p < 0.05 and ** p < 0.01.

In the western blotting of proteins extracted on Day 4, the relative levels of phosphorylated ERK1/2 and AKT, normalized to total ERK1/2 and AKT, were analyzed. The relative phosphorylation of ERK1/2 was significantly increased in the Change group compared with that in the Control group (p = 0.027 and p = 0.015, respectively). In the Low pH group, ERK1 phosphorylation was significantly increased compared with that in the Control group (p = 0.018); however, ERK2 phosphorylation showed no significant differences (p = 0.066). Similarly, the relative phosphorylation of AKT was higher in the Low pH and Change groups than in the Control group (p = 0.026 and p = 0.001, respectively). No significant differences in HIF-1α expression were observed among the groups (Fig 6).

### Suppression of ERK1/2 and AKT during daily 20-min low-pH exposure inhibited the proliferation and tube formation of the HUVECs

**Fig 7:**
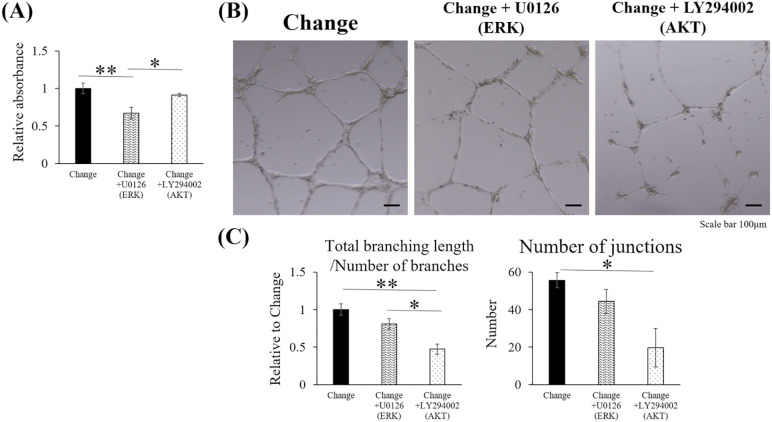
Daily exposure to the low-pH medium containing ERK1/2 and AKT inhibitors suppressed the proliferation and tube formation activities of the HUVECs. Cell proliferation and tube formation were evaluated under conditions with or without pharmacological inhibitors in the low-pH medium during the 4-day pH change experiment. (A) Cell proliferation was measured using the CCK-8 assay (n = 5). (B) Representative microscopic images of the tube formation assay. Scale bar: 100 μm. (C) Quantitative analysis of the total branching length, number of branches, and number of junctions (n = 3). Change: condition of the Change group without pharmacological inhibitors. Change + U0126: condition of the Change group with the ERK1/2 inhibitor (U0126) in the low-pH medium. Change + LY294002: condition of the Change group with the AKT inhibitor (LY294002) in the low-pH medium. Data are presented as the mean ± standard error. Statistical comparisons were performed using a one-way ANOVA, followed by Tukey’s *post-hoc* test. * p < 0.05 and ** p < 0.01. Quantitative analysis was performed using ImageJ software.

To demonstrate that the cellular activities of the HUVECs were upregulated through the ERK1/2 MAPK and PI3K/AKT pathways, cell proliferation and tube formation were analyzed under conditions with or without the pharmacological inhibitors of ERK1/2 (U0126) or AKT (LY294002) in the low-pH medium during the pH change experiment. The proliferation assay using CCK-8 showed lower absorbance in the group treated with U0126 than in the untreated group (p = 0.006), whereas no significant difference was observed with or without LY294002 (Fig 7A). In the tube formation assay, the total branching length per number of branches and number of junctions were decreased in the group treated with LY294002 compared with those in the untreated group (p = 0.005 and p = 0.032, respectively). No significant difference was observed with or without U0126 (Fig 7B, 7C).

## Discussion

The primary findings of this study are that daily intermittent exposure to a low-pH medium for 20 min significantly enhanced the tube formation and migration abilities of HUVECs compared with the effects of the control condition. Additionally, these conditions increased the protein expression of VEGFR2 and VEGF and the mRNA expression of CD31 while activating ERK1/2 and AKT signaling by elevating their phosphorylated levels. The suppression of ERK1/2 and AKT by their inhibitors in the low-pH medium during the pH change experiment inhibited the proliferation and tube formation of HUVECs, indicating that the ERK1/2 MAPK and PI3K/AKT pathways are the primary mediators of cellular activation in response to intermittent acidic stimulation.

Previous reports have shown that acidic pH activates various cell types, including endothelial cells [15,16,23], neutrophils [24], malignant tumor cells [25,26], and chondrocytes [27]. We have emphasized the angiogenic effect of transcutaneous CO_2_ therapy through the Bohr effect [1–5]. Endothelial cells are crucial in angiogenesis during bone and muscle regeneration [6,28]. Therefore, in this study, we focused on endothelial cells to investigate the effects of pH changes on their activities.

An acidic environment activates the ERK1/2 MAPK and PI3K/AKT pathways in endothelial cells [15] and other cell types [18,24,25]. MAPKs are significant in the transcriptional regulation of VEGF and controlling the proliferation and growth arrest of vascular endothelial cells at confluency [29]. The PI3K/AKT pathway participates in fundamental endothelial functions, including the regulation of vascular tone, promotion of angiogenic potential, control of cell adhesion, and recruitment of leukocytes to the vessel wall [30]. Based on previous studies, it is suggested that these pathways contribute to the proangiogenic effects induced by pH changes; therefore, we examined the protein expression of phosphorylated ERK1/2 and AKT using western blotting. Additionally, to evaluate the activation of the VEGF pathway induced by intermittent pH changes in HUVECs, we analyzed the expression of VEGF and VEGFR2. Endothelial cells primarily express the VEGF receptor and may produce VEGF. This cell-autonomous activation of the VEGF signaling pathway contributes to the maintenance of vascular homeostasis [31].

In the Low pH group, proliferation was reduced compared with that in the Change group, but showed no significant difference from the Control group. Nevertheless, tube formation was enhanced, CD31 expression increased, and phosphorylated ERK1 and AKT levels were elevated compared with those in the Control group. Various reports have described the response of endothelial cells to acidic pH. These responses depend on the conditions of acidic exposure and the cell type. CD34-positive endothelial colony-forming cells incubated at pH 6.6 for 24 h showed a significant increase in tube formation, whereas their proliferation and migration were not significantly different compared with those of cells incubated at pH 7.4 [15]. Molar dental pulp endothelial cells incubated at pH 5.4 and 6.4 for 3 days showed significantly increased tube formation compared with the event in those incubated at pH 7.4 [23]. These results are similar to our present findings; however, the incubation conditions and time points differ. Conversely, bovine aortic endothelial cells incubated at pH 7.0 exhibited reduced proliferation compared with that of cells incubated at pH 7.4 (consistent with our findings) but showed decreased tube formation ability (contradicting our results) [13]. Another report showed that HUVECs incubated at low pH exhibited reduced proliferation (aligning with our findings) but decreased migration ability compared with those under normal conditions (inconsistent with our results) [14]. In our study, the slight difference in medium pH between the Low pH and Control groups compared with that in these studies might have influenced the inconsistent results.

Conversely, we defined the Change group as that where the medium was replaced with a low-pH medium for 20 min once daily, and this procedure was repeated for several days per our transcutaneous CO_2_ therapy protocol [1–3]. In the Change group, although proliferation showed only a slight, non-significant increase, tube formation and migration were markedly enhanced, accompanied by elevated CD31, VEGFR2, VEGF, and phosphorylated ERK1/2 and AKT expression. While some studies have reported on acidic preconditioning, studies investigating the effects of repeated acidic preconditioning are lacking. One study reported that CD34-positive endothelial colony-forming cells, initially incubated at pH 6.6 and subsequently returned to pH 7.4, showed increased proliferation [15]. Similarly, human melanoma cells initially incubated at pH 6.8 for 48 h and subsequently returned to pH 7.4 exhibited enhanced proliferation, tube formation, and invasiveness with an upregulated VEGF expression [26]. These findings imply that repeated acidic preconditioning may enhance cellular activities, as demonstrated in our study.

Our findings suggest that the ERK1/2 and AKT pathways are associated with the molecular mechanism of repeated acidic preconditioning. Some reports on endothelial cells have shown that acidic preconditioning promotes these pathways [15,32], and similar results have been observed in ischemic myocardium [18] and neutrophils [24]. In studies on preconditioning with factors other than acidosis, research focusing on enhancing the activity of mesenchymal stem cells is relatively common. Preconditioning with cytokines such as Stromal cell-derived factor 1 (SDF-1) and Tumor necrosis factor-alfa (TNF-α) activates mesenchymal stem cells through the ERK1/2 and AKT pathways [33,34]. SDF-1 is induced by hypoxia and myocardial ischemic preconditioning [35], whereas TNF-α is transiently elevated in response to bone fractures [34]. Considering that intratissue acidosis is induced under these conditions, our findings align with these reports and provide supportive evidence for our study.

Our results showed that HIF-1α expression was not upregulated under conditions of sustained low pH or intermittent acidic preconditioning. Conflicting reports exist regarding the activation of HIF-1 under acidic conditions. HIF-1α is activated at pH 7.0 in endothelial cells [36]; however, acidosis-induced VEGF upregulation is independent of HIF-1α [13]. CO_2_ treatment of HUVECs activated inactive ERK1/2 while inhibiting active ERK1/2 and suppressing HIF-1α expression [37]. Although therapeutic CO_2_ application promotes angiogenesis, it reduces or prevents HIF-1α production [38]. Furthermore, the stabilization of HIF-1α and HIF-2α proteins was reduced by artificially lowering extracellular pH [39]. While hypoxic preconditioning activates HIF-1α [40], our findings suggest that acidic preconditioning in the absence of hypoxia promotes angiogenesis through a pathway independent of HIF-1α.

This study had some limitations. First, during transcutaneous CO_2_ treatment, the CO_2_ concentration and extracellular pH levels at the treatment site were not measured directly. In our previous report, NMR demonstrated that the pH levels in the triceps surae muscle of healthy human volunteers decreased to approximately 7.0 during this therapy [5]. We used this value as a reference in the present study. However, the pH measured using NMR primarily reflects intracellular rather than extracellular pH and has low sensitivity for detecting subtle pH changes in living systems [41]. Extracellular pH is expected to decrease in parallel with intracellular pH [42], and this may also occur during transcutaneous CO2 therapy; however, *in vivo* monitoring is required to confirm this. More accurate techniques for measuring physiological pH (intracellular and extracellular), such as fluorescence imaging, have been developed recently [43]. If such technologies could be applied to monitor pH changes at the CO_2_ treatment site *in vivo*, future studies could be conducted in a manner that more closely reflects a clinical setting. Second, the pH value of the medium is affected by the incubation environment and duration. The low-pH medium was prepared in the same way using the same incubator, and its pH was confirmed before the experiment to standardize this. Nevertheless, the medium pH may vary during the experimental intervention.

Daily 20-min intermittent low-pH stimulation enhanced the tube formation and migration abilities of HUVECs, along with increased expression of CD31 mRNA and VEGFR2 and VEGF proteins. The ERK1/2 MAPK and PI3K/AKT pathways may participate in the cellular response to intermittent pH change stimulation. Our findings could help clarify the molecular mechanisms underlying increased blood flow and angiogenesis during transcutaneous CO_2_ application, which previously remained unclear. For an enhanced understanding, further steps would include detailed monitoring of intratissue pH during *in vivo* CO_2_ therapy. Therefore, we aim to advance the treatment to the clinical phase.

## Supporting information

S1 WB_raw_images. (PDF)
