# Supplementary material for: Effect of intermittent extracellular low-pH environment on human umbilical vein endothelial cell activity
Source: PLoS One. 2025 Sep 16;20(9):e0332673. doi: 10.1371/journal.pone.0332673 (PMC12440188; doi:10.1371/journal.pone.0332673)
Supplement: S1 — (PDF) [file pone.0332673.s001.pdf]

Figure. 5

(B)

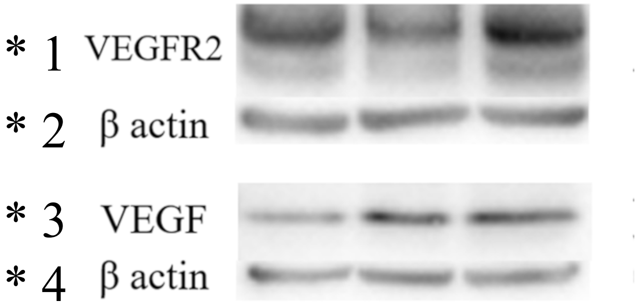

1: VEGFR2

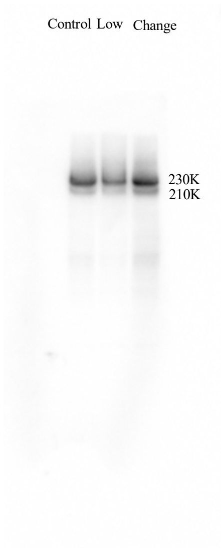

2: β-actin (VEGFR2)

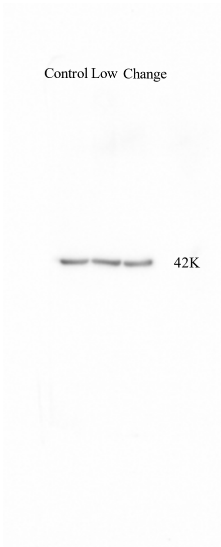

3: VEGF

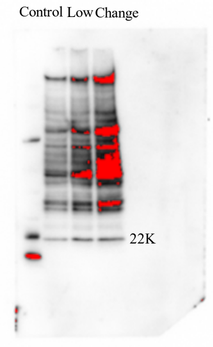

4: β-actin (VEGF)

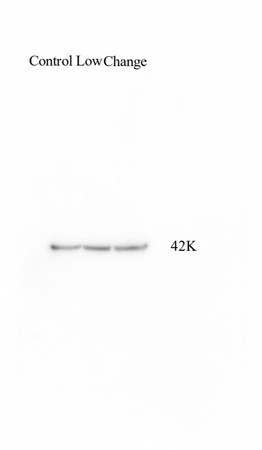

Figure. 6

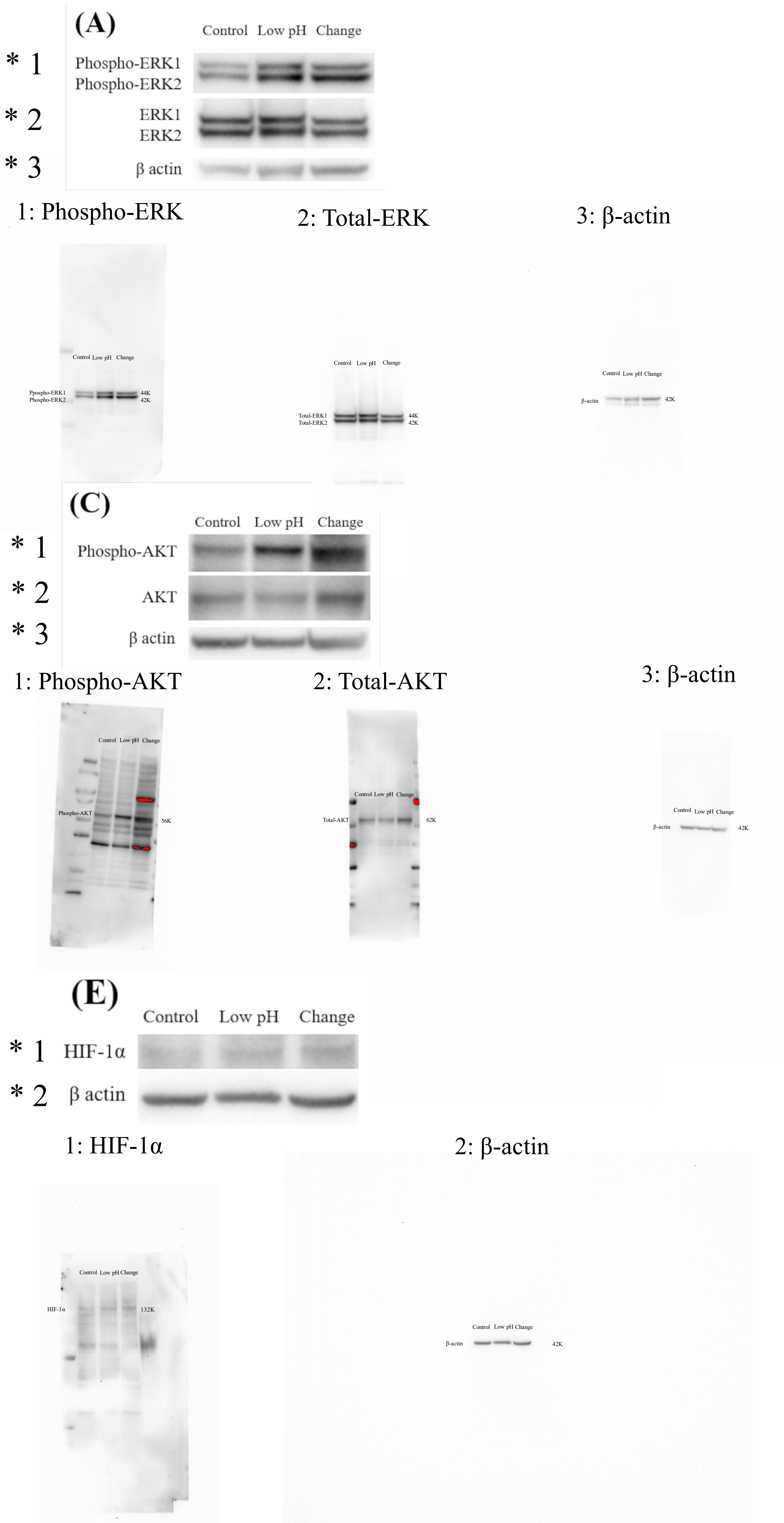
(A)
Control
Low pH
Change
* 1
Phospho-ERK1
Phospho-ERK2
* 2
ERK1
ERK2
* 3
β actin
1: Phospho-ERK
2: Total-ERK
3: β-actin
Ppospho-ERK1
Phospho-ERK2
44K
42K
Total-ERK1
Total-ERK2
(C)
Phospho-AKT
AKT
1: Phospho-AKT
2: Total-AKT
56K
Total-AKT
62K
(E)
HIF-1α
1: HIF-1α
2: β-actin
132K
